# Impact of Nutritional Changes on Nonalcoholic Fatty Liver Disease

**DOI:** 10.3390/nu11030677

**Published:** 2019-03-21

**Authors:** Carolina M. Perdomo, Gema Frühbeck, Javier Escalada

**Affiliations:** 1 Department of Endocrinology and Nutrition, Clínica Universidad de Navarra, 31008 Pamplona, Spain; cperdomo@unav.es (C.M.P.); gfruhbeck@unav.es (G.F.); 2 CIBER Fisiopatología de la Obesidad y Nutrición (CIBERobn), ISCIII, 28029 Madrid, Spain

**Keywords:** NAFLD, NASH, diet, macronutrients

## Abstract

Non-alcoholic fatty liver disease (NAFLD) is a major global health threat due to its growing incidence and prevalence. It is becoming the leading cause of liver disease in addition to its strong association with cardio-metabolic disease. Therefore, its prevention and treatment are of strong public interest. Therapeutic approaches emphasize lifestyle modifications including physical activity and the adoption of healthy eating habits that intend to mainly control body weight and cardio-metabolic risk factors associated with the metabolic syndrome. Lifestyle interventions may be reinforced by pharmacological treatment in advanced stages, though there is still no registered drug for the specific treatment of NAFLD. The purpose of this review is to assess the evidence available regarding the impact of dietary recommendations against NAFLD, highlighting the effect of macronutrient diet composition and dietary patterns in the management of NAFLD.

## 1. Introduction

Non-alcoholic fatty liver disease (NAFLD) results from hepatic fat accumulation (>5% of liver weight), which is not due to excess alcohol consumption, autoimmune, infectious or other established liver diseases [1,2]. NAFLD can exist as pure steatosis, steatosis with mild lobular inflammation, non-alcoholic steatohepatitis (NASH) and hepatocellular carcinoma. In addition to the degree of fibrosis, in the liver biopsy NASH is classified as mild fibrosis (F0–F1), significant fibrosis (≥F2), advanced fibrosis (≥F3, bridging) and cirrhosis (F4). No accurate data exist on the incidence of NAFLD [3]. The worldwide prevalence of NAFLD ranges between 8–45%. In North America and Europe, it is estimated to range from 25–34% and, in Asia, between 15–20%. The highest prevalence is described in the Middle East and South America. However, in certain subpopulations (i.e., obesity, type 2 diabetes [T2D]) the estimated prevalence of NAFLD is significantly higher, ranging from 43–92%. When compared to the general population, patients with NAFLD have higher mortality from both liver and non-liver-related causes. They are at increased risk for cardiovascular diseases [4], T2D and chronic kidney disease [2]. NAFLD is currently ranked as the second most common cause of liver transplantation and is predicted to become the first in Western countries [3]. Additionally, NAFLD is the second most common cause of hepatocellular carcinoma. Therefore, NAFLD is a major global health threat; its prevention and treatment represent a mounting challenge in health services. Therapeutic approaches focus on lifestyle modification [5]. Diet and exercise interventions remain as the first line of therapy, aiming mainly at controlling body weight and cardio-metabolic risk factors related to metabolic syndrome. In the early stages of NAFLD, a healthy diet and weight loss of at least 7% might be sufficient [4]. In more advanced stages, high genetic risk or, in the presence of diabetes, intensified lifestyle intervention reinforced by pharmacological treatment might be necessary, though there is still no registered drug for the treatment of NAFLD. This review assesses the evidence available for dietary recommendations against NAFLD.

We searched for original articles and reviews published between 1 January 2000 and 31 December 2018, focusing on NAFLD dietary treatment in PubMed and MEDLINE using the following search terms (or combination of terms): “NAFLD”, “NASH”, “fatty liver”, “macronutrient”, “dietary”, “recommendations”, “composition”, “life-style intervention”, “Mediterranean diet”. Only full-text articles written in English were included. More weight was given to studies with a high level of evidence from either randomized controlled trials, prospective case-control studies, meta-analyses or systematic reviews. Articles in journals with explicit policies governing conflicts of interest and stringent peer-review processes were favored. Data from larger replicated studies with longer periods of observation when possible were systematically chosen to be presented. Reference lists of retrieved articles were used to obtain additional references.

## 2. Pathophysiology

The major established risk factors for NAFLD include obesity, insulin resistance and dyslipidemia [5]. Genetic modifiers are associated with the increased progression to NASH and cirrhosis, however, some of them are associated with apparent protection from cardiovascular diseases [4]. The best-characterized genetic association is with *PNPLA3* [6] encoding I148M (regulator of the mobilization of triglycerides from lipid droplets) and with TM6SF2 [1,3,4] encoding E167K (regulator of very low-density lipoprotein (VLDL) secretion).

Dietary lipids (15%), lipolysis of adipose tissue (60–80%) and de novo lipogenesis (5%) contribute to the pool of lipids stored in the liver [7]. Adipose tissue lipolysis is regulated by the actions of insulin on adipocytes [2,8]. *De novo* lipogenesis is the process in which hepatocytes convert excess carbohydrates, especially fructose, into fatty acids [2], it is strictly regulated by nuclear receptor and cytoplasmic transcription factors including the liver X receptor (intervening in hepatic fatty acid synthesis), the farnesoid X receptor (interfering in VLDL assembly) and the peroxisome proliferator-activated receptors [PPARs] family [7]. PPAR-α regulates free fatty acids oxidation, PPAR-γ has an anti-inflammatory function, and PPAR-δ suppresses hepatic lipogenesis and reduces the hepatic expression of pro-inflammatory genes.

Lipid removal is mediated by both mitochondrial fatty acid β-oxidation and re-esterification to form triglyceride [7]. Triglycerides can be exported into the blood as VLDL or may be stored in lipid droplets. Lipid droplet triglycerides may undergo lipolysis to release fatty acids back into the hepatocyte free fatty acid pool. In order to understand the different nutritional advice for the treatment of NAFLD, it is necessary to comprehend that insulin resistance in adipose tissue contributes to fat accumulation and NASH through dysregulated lipolysis, resulting in the excessive delivery of fatty acids to the liver.

A ‘two-hit’ hypothesis to explain NAFLD development has been proposed. The ‘first hit’ is steatosis, the accumulation of fat in hepatocytes because of excessive triglycerides due to an imbalance in the lipid metabolism [7,9]. Steatosis is a consequence of insulin resistance [5,10,11]. Insulin resistance leads to hepatic triglyceride accumulation due to the increase of lipolysis in the peripheral adipose tissue resulting in higher levels of circulating non-esterified fatty acids, which are taken up by the liver and esterified into triglycerides. Impaired insulin signaling results in compensatory hyperinsulinemia [5]. Hyperinsulinemia decreases glycogen synthesis, which increases the hepatic fatty acid uptake, alters triglycerides transportation, and inhibits liver β-oxidation. In addition, glucose can be taken up by the liver through an insulin-independent transporter and be converted to pyruvate (a precursor of acetyl-CoA and malonyl-CoA) which can be transformed into fatty acids through de novo lipogenesis [5,10]. All of these alterations increase the pro-inflammatory cytokine activity leading to oxidative stress-mediated lipotoxicity, impaired hepatocyte apoptosis, inflammasome activation and mitochondrial dysfunction, contributing to fatty acid accumulation, hepatocellular injury, inflammation and the progressive accumulation of excess extracellular matrix [2]. Oxidative stress, triggered by pro-inflammatory cytokines (such as tumor necrosis factor-alpha [TNF-α], interleukin-6 and interleukin-8) or the reduction of anti-inflammatory cytokines (adiponectin), is considered the ‘second hit’ of NAFLD’s pathogenesis [11]. This may further exacerbate insulin resistance and hepatocyte injury induced by genetic or environmental susceptibilities.

**Figure 1:**
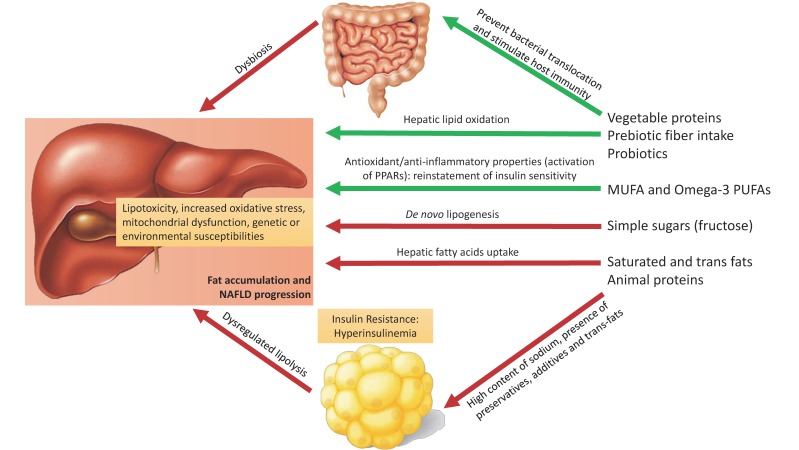
The effects of diverse macronutrients on non-alcoholic fatty liver disease pathophysiology. An unhealthy dietary pattern including saturated fats, trans fats, simple sugars and animal protein (red and processed meat) results in an increased total and visceral fat mass, insulin resistance, increased hepatic de novo lipogenesis and gut dysbiosis. Under these conditions and acting parallel, fat accumulates in the liver causing lipotoxicity, increased oxidative stress and mitochondrial dysfunction adding genetic or environmental predisposition for hepatic lipid accumulation (‘multiple hit hypothesis´). Red arrow: unfavorable/harmful effect; Green arrow: favorable/beneficial effect. NAFLD: non-alcoholic fatty liver disease; PPAR: peroxisome proliferator-activated receptors.

A ‘three-hit’ hypothesis has been proposed as a result of inadequate hepatocyte regeneration and apoptosis [9]. However, all of these hypotheses might be outdated because free fatty acid accumulation alone is sufficient to induce liver damage [2]. It is not even certain whether NASH is always preceded by steatosis. The variable progression of NAFLD has led to the description of a ‘multiple-hit’ hypothesis suggesting that multiple events act in parallel (including lipotoxicity, proinflammatory cytokines, increased oxidative stress, mitochondrial dysfunction, genetic or environmental susceptibilities) [9,11]. Consequently, in NAFLD, a modest weight reduction through diet and exercise leads to an improvement in the substrate overload and insulin sensitivity [12]. Figure 1 summarizes the liver effects of the different macronutrients present in the diet composition (discussed ahead).

## 3. Lifestyle Intervention

A 2011 systematic review (23 trials) [13] assessed the effect of diet and physical activity on NAFLD. Lifestyle modifications leading to weight reduction and/or increased physical activity consistently showed reductions in liver fat, aminotransferase concentrations and improved insulin sensitivity. The strongest correlation was seen with a weight reduction of >7%. A reduction in inflammation was evidenced in five trials that included histopathology. However, only one study revealed a significant fibrosis reduction. A recent network meta-analysis (19 studies) [12] confirmed that in NAFLD, exercise plus a dietary intervention is the most effective treatment. Dietary intervention may be more effective in improving aminotransferases, although exercise showed superiority in improving insulin sensitivity and in reducing body mass index (BMI). Look AHEAD (Action for Health in Diabetes) [14] is a multicenter clinical trial including 5145 overweight adults with T2D. Steatosis reduction was evidenced after a 12-month intensive lifestyle intervention, achieving a weight loss of at least 7%. A recent randomized controlled trial (*n* = 154) [15] reaffirmed that lifestyle intervention with a weight reduction in a dose-dependent manner effectively leads to NAFLD remission in non-obese and obese patients. However, weight loss and maintenance is a difficult task. There is an urgent need to simplify dietary recommendations for NAFLD patients.

## 4. Diet Composition Focusing on Macronutrients

Independently of energy intake, the macronutrient composition of a diet is associated with NAFLD/NASH development [16]. Different epidemiological studies have identified macronutrients with a harmful or beneficial effect on the liver (Table 1). Since 2003, Musso et al. [17] identified that dietary habits may directly promote NASH by modulating hepatic triglyceride accumulation and antioxidant activity and, indirectly, by affecting insulin sensitivity and the postprandial triglyceride metabolism. They examined 25 NASH patients vs. 25 healthy controls. Compared to controls, patients with NASH had a significantly higher saturated fat intake and a smaller amount of polyunsaturated fatty acid (PUFAs), fiber, vitamin C and vitamin E consumption. Similarly, in 2006, Cortez-Pinto et al. [18] compared the dietary composition in 45 NASH patients vs. 856 healthy controls. NASH patients had a significantly higher total fat and omega-6 PUFAs consumption and a lower fiber intake. In 2014, a cross-sectional Korean study in 348 patients (169 NAFLD patients vs. 179 healthy controls) [19] found an inverse association between NAFLD risk and vitamin C, vitamin K, folate, omega-3 PUFAs, and nuts and seeds consumption. In 2016, Wehmeyer et al. [20] examined 55 NAFLD patients vs. 88 healthy controls. NALFD patients had a higher energy intake with a significantly higher glucose and protein consumption and a lower fiber and mineral consumption per 1000 kcal. However, no significant differences regarding carbohydrates, fructose and fat per 1000 kcal energy intake were appreciated. Recently, the macronutrient composition was analyzed in NAFLD patients between 61–79 years of age from the Rotterdam study [21]. A total of 3882 participants were included (NAFLD patients = 1337). The total protein intake was associated with NAFLD in overweight patients even after adjusting for sociodemographic and lifestyle covariates. However, after the adjustment for metabolic covariates, only animal proteins remained associated. Monosaccharides, disaccharides and fiber were not associated with NAFLD.

Overall, by being independent of weight loss and energy intake, a healthy diet is effective in reducing liver fat content and, therefore, protecting one from cardio-metabolic morbidity and mortality [4]. Contemporary dietary recommendations for NAFLD treatment include caloric restriction and adherence to the macronutrient composition of the Mediterranean diet (MD) [1]. However, before recommending a specific dietary pattern, it is necessary to analyze the liver effect of each macronutrient.

### 4.1. Fats

#### 4.1.1. Saturated Fats

Saturated fats are generally found in animal products (red meat, cream, butter and whole milk dairy products), some vegetable products (coconut oil, palm oil and palm kernel oil) and prepared foods (desserts and sausages) [16]. Saturated fat intake is correlated with an impaired glutathione metabolism towards oxidative stress leading to NAFLD progression. A meta-analysis (8 randomized trials) [22] including 13,614 patients found a 10% risk reduction of coronary events (myocardial infarction and/or cardiac death) for every 5% of energy intake conferred by PUFAs after replacing saturated fats for at least 1 year in the general population. Interestingly, mice fed with saturated fats vs. regular chow-diet had a *PNPLA3* expression in hepatocytes that was 23 times higher [23]. This upregulation was reversed with fasting. In individuals that consume high saturated fat diets, genetic variations may influence NAFLD susceptibility. Overall, it is reasonable to suggest a saturated fat intake reduction in the general population.

#### 4.1.2. Monounsaturated Fats

Monounsaturated fatty acids (MUFAs) are found in olive oil, avocados and nuts [24]. Olive oil is the most representative ingredient in the traditional MD. Olive oil is predominantly constituted by MUFAs (70–80%) and palmitic acid (up to 20%). Higher grades of olive oil (e.g., extra-virgin and virgin) contain higher amounts of polyphenols and phytochemicals that are lost when olive oil is refined or heated. Phenolic compounds in MUFAs have shown antioxidant and anti-inflammatory properties that may induce an improvement in dyslipidemia and endothelial dysfunction [25]. A decreased risk of metabolic syndrome and/or cardiovascular disease has been evidenced with a higher MUFAs consumption [24,25]. Additionally, dietary MUFAs may be protective against age-related cognitive deterioration, Alzheimer’s disease and cancer [25]. A systematic review (9 trials) [26], including 1547 T2D patients, evaluated the effect of MUFAs in glycemic control. A significant Hb1Ac reduction was evidenced without fasting plasma glucose or homeostatic model assessment of insulin resistance (HOMA-IR) improvement.

Regarding NAFLD, in 2006, Cortez-Pinto et al. [18] revealed a higher MUFAs consumption in patients with NAFLD. Similarly, in 2017, Rietman et al. [27] assessed the dietary intake in 1128 patients and established an association with the Fatty Liver Index (FLI; derived from BMI, waist circumference, triglycerides and gamma-glutamyltransferase [GGT]). MUFA consumption and total fat intake were positively associated with a higher FLI score. In the Rotterdam Cohort [21], MUFA consumption did not suggest a beneficial effect in NAFLD.

On the other hand, in 2007, Zelber Sagi et al. [28] did not find an association between MUFAs and NAFLD development. A randomized parallel-group controlled trial (*n* = 45) [29] in patients with T2D showed a major liver fat reduction (measured by proton nuclear magnetic resonance spectroscopy (1H NMR)) following an 8-week isocaloric MUFAs enriched diet vs. a high fiber and low glycemic index carbohydrate diet, independently of an aerobic training program. Additionally, a recent randomized, double-blind clinical trial (*n* = 66 NAFLD patients) [30] evaluated the effect of 20 g/day of olive oil vs. sunflower oil for 12 weeks. Olive oil improved fatty liver severity (measured by ultrasonography (US)), triglyceride levels and fat mass independently of correcting cardio-metabolic risk factors. Both MUFAs and PUFAs might reduce steatosis in NAFLD by PPARs activation, which stimulates free fatty oxidation and decreases inflammation, insulin resistance and the expression of genes involved in hepatic de novo lipogenesis [16]. While there is still controversy in some studies, it is prudent to recommend moderate MUFAs consumption (20 g/day).

#### 4.1.3. Polyunsaturated Fats

Essential PUFAs includes omega-3 PUFAs and omega-6 PUFAs. The latter are mostly found in vegetable oils (canola and cottonseed), cereal grains (wheat, maize and rice) and nuts [18,31]. Linoleic acid is the main dietary omega-6 PUFAs. Increased amounts of omega 6-PUFAs are related to a higher incidence of inflammatory and thrombotic events, including cardiovascular disease, cancer, inflammatory and autoimmune diseases. An omega-6/omega-3 ratio of 15:1 has been described in the Western diet [31]. Nevertheless, in a randomized trial (*n* = 67), lower insulin levels, inflammatory markers and reduced hepatic steatosis (assessed by magnetic resonance (MRI) or 1H NMR) were evidenced after a 10-week isocaloric diet supplemented with omega-6 PUFAs vs. saturated fat supplementation [32].

Omega-3 PUFAs are mostly found in seafood (pilchards, sardines, mackerel, trout, salmon, herring and tuna, haddock, cod, and crustaceans, shellfish), in certain vegetable oils (flaxseed oil) and, in lower amounts, in eggs and meat [10]. Marine Omega-3 PUFAs include eicosapentaenoic acid (EA), docosahexaenoic acid (DHA) and docosapentaenoic acid. Omega-3 PUFAs modulate hepatic lipid composition and increase anti-inflammatory mediators leading to an improvement of insulin sensitivity that induces triglyceride redistribution (lipid storage in adipose tissue and the consequent decrease of triglyceride serum levels) [5,10].

In a cohort of 12,300 patients with multiple cardiovascular risk factors and after a 5-year follow-up, daily treatment with omega−3 PUFAs did not reduce cardiovascular events or mortality [33]. Yet, a systematic review (23 randomized controlled trials) [34] evaluated the effect of omega-3 PUFA supplementation in 1075 T2D patients with cardiovascular risk factors. Omega-3 PUFA supplementation (mean dose: 3.5 g/day; mean treatment duration: 8.9 weeks) showed improved dyslipidemia (triglyceride and VLDL cholesterol serum levels), with no statistically significant effect on body weight, glycemic control, fasting insulin, total-cholesterol or HDL-cholesterol.

Regarding NAFLD, in 2004, Araya et al. [35] used liquid gas chromatography to analyze liver and abdominal adipose tissue fatty acids in 19 patients with NAFLD vs. 11 controls; a marked enhancement in omega-6/omega-3 ratio characterized the liver of NAFLD patients. The first report about omega-3 PUFAs supplementation in NAFLD patients was performed in 2006 [36]. Forty-two NAFLD patients received 1 g daily of EA and DHA (in the ratio of 0.9:1.5 respectively) for 12-months vs. 14 controls. Omega 3-PUFAs supplementation significantly decreased the serum GGT, aspartate aminotransferase (AST), alanine aminotransferase (ALT), triglycerides serum levels and fasting glucose. Additionally, the circulating arachidonate and omega-6/omega-3 ratio were reduced in treated patients. Moreover, US demonstrated the improvement of liver echotexture and the increase of the Doppler perfusion index after omega-3 supplementation without significant changes in the control group. A study found a total PUFAs intake below the recommended level and lower hepatic omega-3 and omega-6 PUFAs in NASH-biopsy-proven patients (*n* = 38) vs. simple-steatosis-biopsy-proven patients (*n* = 18) [37]. Patients with NASH had a higher BMI, central obesity, body fat, insulin resistance, dyslipidemia and lower physical activity. As suspected, higher liver lipid peroxides with a lower antioxidant power were evidenced, addressing the higher oxidative stress that leads to the metabolic abnormalities. Similarly, a Japanese study [38] evidenced a lower PUFAs/saturated fats intake ratio in biopsy-proven NASH patients (*n* = 28) vs. simple-steatosis-biopsy-proven patients (*n* = 18).

In 2008, two studies investigated the effect of calorie-restricted diets with and without PUFA consumption. Spadaro et al. [39] found a significant improvement in ALT, triglyceride and TNF-α serum levels, as well as an improvement in the fatty liver measured by US after 2 g/day PUFAs and a 25–30 kcal/kg/day diet in NAFLD patients (*n* = 20) vs. a control group (*n* = 20) who followed the same diet without PUFAs administration. Zhu et al. [40] proved the efficacy of PUFAs supplementation (seal oil) in NAFLD in a randomized placebo-controlled trial. Seventy-two patients received 2 g of seal oil three times per day plus dietary recommendations vs. 72 controls. PUFAs from seal oil significantly improved their total symptom score, fatty liver severity measured by US, ALT and triglycerides serum levels. In 2012, a meta-analysis (9 studies) [41] of omega-3 PUFAs supplementation including 355 NAFLD patients, suggested an improvement in liver fat and in AST levels, however, substantial heterogeneity was found and an optimal dose was not clarified.

A reduced liver fat measured by US, improved HOMA-IR and reduced triglycerides serum levels without weight gain was evidenced after a 6-month treatment with 250 mg/day (*n* = 20) or 500 mg/day (*n* = 20) of DHA in children [42]. A similar randomized placebo-controlled trial was repeated in the adult population from the WELCOME study (Wessex Evaluation of Fatty Liver and Cardiovascular markers in NAFLD with Omacor therapy) [43]. Sixty patients with NAFLD received omega-3 PUFAs supplementation (4 g of DHA plus EA) for 15–18 months. Reduced liver fat measured by 1H NMR could not be significantly evidenced nor could a reduction in the validated liver fibrosis scores. However, the erythrocyte percentage DHA enrichment using gas chromatography was linearly associated with the decreased liver fat percentage. Likewise, a phase 2b multicenter, double-blinded, randomized, placebo-controlled trial found no histological or blood markers improvement in 243 NASH patients receiving 1.8 g/day or 2.7 g/day of EA vs. placebo for 12 months [44]. However, with 2.7 g of EA, reduced levels of triglyceride were significantly observed.

Flaxseed oil is another source of omega-3 PUFAs. A two-arm randomized clinical trial [45] showed a significantly higher reduction in body weight, liver enzymes, insulin resistance, hepatic steatosis and fibrosis scores after 12 weeks with 30 g/day of flaxseed oil plus lifestyle modification (*n* = 25) vs. lifestyle modification only (*n* = 25). However, no histological improvement was seen after a six-month use of flaxseed oil and fish oil vs. mineral oil in NASH patients despite triglyceride levels significantly improving in flaxseed oil and fish oil arm [46].

As evidenced, few clinical trials have addressed the potential benefits of omega-3 PUFAs on NAFLD/NASH. Larger well-designed clinical trials for longer periods are still needed to fully evaluate the beneficial effects of omega-3 PUFAs in NAFLD. Overall, omega-3 PUFAs is preferable than other forms of PUFAs, therefore, it is essential to recommend omega-3 PUFAs intake with an omega-6/omega-3 fatty acid ratio of about 1–2:1 [31].

#### 4.1.4. Trans-Fats

The substantial loss of trans-fats from the liver lipid pool in parallel with NASH improvement suggests a potential role of trans-fats in the pathogenesis of NAFLD/NASH. Alferink et al. [21] recently indicated that trans-fats (predominantly from desserts, cream or solid fats) were associated with higher odds for NAFLD. Due to known trans-fat harmful effects in humans, there is a lack of human clinical trials evaluating the effect on the liver. In an animal study [47], mice were fed with a diet high in trans-fats (partially hydrogenated vegetable oil) and fructose corn syrup for 16 weeks. They developed hyperinsulinemia and severe hepatic necroinflammation. However, when trans-fats were removed, NASH improved biochemically and histopathologically [48]. Moreover, hepatocellular carcinoma development was observed in 6 out of 10 mice after a 12-month high dietary trans-fats and fructose corn syrup intake plus a sedentary lifestyle [49]. Without hesitation, it is necessary to advise minimizing or avoiding trans-fats consumption.

### 4.2. Protein

Regarding the protein content in diet, several studies have evidenced a significantly higher protein intake in patients with NAFLD [18,20,28,50]. However, other studies have found no difference in protein intake in patients with NAFLD compared to controls [17,51]. Controversies may be explained by the nature of the protein consumed. Rietman et al. [27] found an inverse association between vegetable protein and NAFLD assessed by FLI score in a Dutch adult population (*n* = 1128). Animal protein, soft drinks and less dietary fiber intake were positively associated with NAFLD.

Bortoletti et al. [52] observed a 20% reduction in intrahepatocellular lipids measured by MR in sedentary obese women that consumed 60 g/day of whey protein for 4 weeks. An improvement in plasma lipid profile was also observed, without effects on glucose tolerance or creatinine clearance. Similarly, soy protein intake improved liver function in mice with NASH [53]. Soy protein has isoflavones, which have an anti-oxidative ability capable of improving insulin resistance and, therefore, lowering lipid levels. Amino acid catabolism requires energy, subsequently, a high protein intake might generate increased hepatic lipid oxidation that could explain the beneficial effect of vegetable protein in NAFLD [16]. Moreover, experimental animal data suggest that taurine, a non-essential amino acid and a bile acid conjugate, may reduce hepatic lipid accumulation, inflammation, triglycerides and insulin serum levels [54]. Taurine supplementation may ameliorate liver injury, henceforward, becoming a possible treatment for diet-induced NAFLD.

On the contrary, it is well known that high meat intake, especially processed meats, is associated with impaired insulin sensitivity and, therefore, may increase the risk of T2D [55,56]. The PREDIMED study [57] has demonstrated in 717 participants (after a year of follow up) that higher red meat consumption is associated with a significantly higher incidence and prevalence of metabolic syndrome. Regarding NAFLD, a cross-sectional study (*n* = 349) [28] determined that NAFLD patients consumed 27% more protein from all types (of high and low-fat meat) including beef, liver, sausage, lamb, chicken and turkey. Similarly, a nested case-control study [58] was conducted with 200 NAFLD patients vs. 200 matched by age and gender controls. NAFLD patients had a significantly higher red meat consumption level. In the Rotterdam cohort [21], NAFLD patients (*n* = 1337) had a higher protein intake even after adjustment for sociodemographic, lifestyle and metabolic covariates vs. controls. As expected, high processed meat consumption is associated with an increased risk of NAFLD due to its high sodium content and the presence of preservatives, additives, food flavor enhancers [59], saturated and trans-fats [56,60].

A recent large epidemiological study [61] from six states and two metropolitan areas in the United States of America (USA) (*n* = 536,969; aged 50–71; 16-year follow-up) revealed that high red meat intake was associated with all-cause mortality and specifically with mortality from liver diseases. They also demonstrated reduced risks after substituting red meats for white meat, particularly unprocessed white meat. Heme iron and processed meat nitrate/nitrite from processed meat were independently associated with mortality. Furthermore, a recent Israeli study (*n* = 357) [62] found that high meat consumption, specifically red and processed meat consumption were associated with higher odds of insulin resistance and NAFLD (assessed by US), independently of saturated fat intake, BMI, physical activity, smoking and alcohol consumption. Additionally, cooking meat at high temperatures for a long duration (fried, grilled or broiled to a well-done level) was independently associated with insulin resistance due to higher intake of heterocyclic amines. A recent German prospective study (*n* = 37) [63] suggested a beneficial effect of either vegetable or animal protein in T2D. Patients were assigned to a diet high in vegetable protein (mainly legume protein) or to a diet high in animal protein (rich in meat and dairy foods) without calorie restriction for 6 weeks. Both diets had the same macronutrient composition (30% protein, 40% carbohydrates, and 30% fat). After 6 weeks, intrahepatic lipid content (assessed by MR and 1H NMR), insulin resistance and hepatic necroinflammation markers decreased. Overall, it seems cautious to limit the consumption of unhealthy meats. Additionally, improved cooking methods should be recommended as part of NAFLD dietary treatment.

### 4.3. Carbohydrates

Several studies have evidenced the detrimental metabolic effects after high consumption of simple carbohydrates (CHO). Wehmeyer et al. [20] observed a significantly higher glucose intake per 1000 kcal in patients with NAFLD (*n* = 55) compared to healthy controls (*n* = 88). A cross-sectional study (*n* = 375 patients) [28] demonstrated that simple CHO consumption (soft drinks) was significantly higher in NAFLD patients. Similarly, a Japanese study [38] exposed that a diet rich in CHO, particularly simple CHO, was associated with a higher risk of biopsy-proven NASH (*n* = 28) vs. biopsy-proven simple steatosis (*n* = 18). Volynets et al. [50] identified a higher intake of total carbohydrates, monosaccharides, disaccharides and protein in NAFLD patients (*n* = 20) vs. controls (*n* = 10). Plasminogen activator inhibitor-1, endotoxin and ALT plasma levels were positively associated with the total protein and carbohydrate intake.

In contrast, in the Rotterdam study [21], the total carbohydrate, monosaccharide and disaccharide intake were inversely related to NAFLD prevalence. The authors advert that this study was performed in an elderly population (61–79 years old) where soft drink consumption was less than 1 drink/day, therefore, fruits were the main source of monosaccharide and disaccharide intake. Similarly, Rietman et al. [27] found that monosaccharide and disaccharide intake was inversely associated with NAFLD, though, soft drink consumption was associated with NAFLD. Dietary fiber is discussed ahead. Overall, the dietary source of monosaccharides and disaccharides is essential to determine their effect on NAFLD.

#### 4.3.1. Fructose

The rising incidence of metabolic syndrome coincides with a marked increase in fructose consumption. In Asian cross-sectional studies, Jia et al. (*n* = 4365) [64] and Tajima et al. (*n* = 2444) [65] reported a positive association between NAFLD risk and high-fructose products (fruit, cakes, soft drinks and sugary snacks). A cross-sectional study [66] from the NASH Clinical Research Network (*n* = 427 NAFLD patients) found that fructose ingestion from soft drinks is associated with more advanced liver fibrosis on biopsy. Evidence suggested that even a 1-week fructose consumption of 4 g/kg/day or a glucose consumption of 3 g/kg/day may increase the liver fat content measured by 1H NMR or MR [67]. A randomized controlled trial [68] evaluated the effect of high sucrose intake (1 L of cola/day for 6 months) vs. water, milk or artificially sweetened soft drink in 47 overweight subjects. An association between sucrose intake and liver fat (measured by 1H NMR) as well as between skeletal fat and visceral fat (measured by dual-energy X-ray absorptiometry and MR) was found. Additionally, higher systolic blood pressure, serum triglycerides and total cholesterol were evident in the sucrose group. In a 4-week double-blind trial (*n* = 32) [69], no differences were found in the HOMA-IR and hepatic fat content (measured by 1H NMR) between high fructose intake vs. high glucose intake. The evidence indicates an energy mediated effect rather than a specific macronutrient effect over fat accumulation. Still, it seems prudent to advise limiting the refined carbohydrate consumption particularly from soft drinks and fruit juices.

It is hypothesized that fructose is a strong de novo lipogenesis inducer, probably due to the direct flow of fructose carbon into the glycolytic pathway, evading phosphofructokinase (a key regulatory enzyme of glycolysis), and thereby contributing to the synthesis of triglyceride glycerol [16]. Additionally, fructose may activate the lipogenic gene expression and may induce bacterial overgrowth in the small intestine which increases the endotoxin levels in the portal vein producing inflammation in NASH.

#### 4.3.2. Dietary Fiber

Poor fiber intake is common in NAFLD [18,20,51,70] and in NASH [17]. The mechanism by which poor fiber ingestion may influence NAFLD has not yet been completely understood [11,71,72]. Some fibers are prebiotics. Prebiotic fibers are a group of non-digestible carbohydrates found in garlic, asparagus, leek, chicory root and onions. A prebiotic is a nonviable food component that confers a health benefit on the host through microbiota modulation. Recently, prebiotic fibers have been added as ingredients to many common food products such as bread, cereal bars and breakfast cereals [11]. Fructooligosaccharides are now becoming increasingly popular due to their prebiotic effect [73]. They promote *Bifidobacteria* as the dominant species in the large bowel, thus, controlling harmful bacteria growth.

Prebiotics modulate the human microbiota by reducing luminal pH and thus inhibiting pathogen growth [11,71,72]. It is known that the translocation of bacteria into the systemic circulation may lead to systemic inflammation that enhances insulin resistance promoting liver injury [74]. However, microbial metabolites, including ethanol and other volatile organic compounds (VOC) produced in a dysbiotic intestinal environment may also have toxic effects on the liver after intestinal absorption [75]. A significantly altered fecal VOC profile and compositional shift in the fecal microbiome is observed in obese patients with clinically suspected NAFLD.

The modification of intestinal bacterial flora is proposed as a therapeutic approach for the treatment of NASH [72]. Animal studies provide promising evidence [11]. Dietary fiber, regardless of fermentability, reduces adiposity and hepatic steatosis in rodents [76]. In humans, a body weight reduction, glucose and lipid metabolism improvement were proven (*n* = 48) after 21 g/day of oligofructose supplementation for 12 weeks vs. placebo [77]. Oligofructose is a form of dietary fiber found in vegetables. A randomized double-blinded cross-over study [78] assessed the effect of prebiotic fiber in seven biopsy-proven NASH patients after receiving, for 8 weeks, 16 g/day of oligofructose vs. placebo. An improvement in the serum aminotransferases and insulin levels was evidenced. Further research is needed to confirm the potential effects of prebiotic fibers in patients with NAFLD, though it is safe and reasonable to promote its consumption.

In summary, macronutrient recommendations for the treatment of NAFLD are mostly based on observational studies and robust research is needed to confirm these findings (Table 2).

### 4.4. Others

#### 4.4.1. Probiotics

Probiotics are bacteria or yeast of the habitual intestinal flora with the capacity of conferring a health benefit on the host [9,71]. Gut microbiota have been implicated in different disorders including metabolic diseases. *Bifidobacterium* and *Lactobacillus* strains are the most commonly used bacteria exhibiting probiotic properties. An increase in *Firmicutes* and a decrease in *Bacteroidetes* has been described in obesity [79].

There has been increasing interest in searching whether people with NAFLD have a dysfunctional microbiome that may promote the NAFLD progression. Intestinal bacteria may be involved in NAFLD by enhancing intestinal permeability, the direct activation of inflammatory cytokines via the release of lipopolysaccharide (LPS), favoring absorption of endotoxins, producing endogenous ethanol, and affecting dietary choline and bile acid metabolism [9,73].

Probiotics communicate with the host immune system through intestinal cell pattern recognition receptors [73,80]. Inefficient antigen presentation and the continuous presence of endotoxin might induce the activation of the innate immune system resulting in chronic subclinical inflammation [9]. In a healthy individual, these bacterial products are rapidly cleared by the hepatic immune system, but in an injured liver, they can result in the release of reactive oxygen metabolites, proteases and other degenerative enzymes, which worsen the liver damage [81]. Dysbiosis in the gut microbiota may prompt the hepatic de novo lipogenesis by increasing the expression of lipogenic enzymes [11]. Moreover, elevated LPS levels have been documented in NAFLD [11,71]. Gut-derived LPS may cross intestine tight junctions inducing liver injury through Kupffer cell activation thus contributing to the onset of liver fibrosis [9,73]. Furthermore, gut microbiota could contribute to producing higher endogenous blood ethanol concentration [9]. Additionally, bile acids are regulators of hepatic lipid and the glucose metabolism and have a strong bactericidal action due to their effect on the bacterial cell membrane phospholipids. The bile acids metabolism can be altered by microbiota and fat-rich diets, contributing to the pathogenesis of NAFLD [9].

Aller et al. [82] investigated the impact of probiotic supplementation in 28 NAFLD adult patients. The probiotic intervention was comprised of 500 million colonies of *Lactobacillus bulgaricus* and *Streptococcus thermophiles* daily for three months. Therapy was associated with a significant reduction in liver enzymes vs. placebo. No significant changes in anthropometrics or cardiovascular risk factors were found between groups. Similarly, a 6-month probiotics trial [83] (*Lactobacillus* and *Bifidobacterium* supplementation) in 10 biopsy-proven NASH patients vs. 10 controls resulted in significantly lower intrahepatic triglyceride (measured by H1 MRS) and AST levels independently of the BMI, waist circumference, glucose or lipid levels. A further trial (*n* = 52 NAFLD patients) [84] receiving the same supplementation for 6 months denoted a reduction in the ALT, AST, GGT, inflammatory markers and liver stiffness assessed by transient elastography vs. placebo.

Vajro et al. [85] examined 22 obese children noncompliant with lifestyle interventions who received either probiotic *Lactobacillus rhamnosus* strain GG (12 billion CFU/day) vs. placebo for 8 weeks. A significant decrease in ALT and in antipeptidoglycan-polysaccharide antibodies irrespective of changes in BMI and visceral fat was observed. In parallel, Malaguarnera et al. [86] evaluated the effects of *Bifidobacterium longum* with fructooligosaccharides and lifestyle modification vs. lifestyle modification alone in 66 patients with biopsy-proven NASH. Probiotic supplementation and lifestyle modification, when compared to lifestyle modification alone, significantly reduced TNF-α, CRP (C reactive protein), AST, HOMA-IR, serum endotoxin, steatosis and the NASH activity index. A 2013 meta-analysis [73] including 4 randomized trials (*n* = 134) suggested that probiotics can reduce insulin resistance, liver aminotransferases, total-cholesterol and TNF-α. However, the use of probiotics was not associated with changes in BMI, glucose and LDL-cholesterol. A review in human randomized clinical trials [71] evaluated the effects of probiotics and synbiotics on obesity, T2D and NAFLD. The beneficial effects of some probiotics and synbiotics improved the liver function and metabolic parameters in patients with NAFLD.

Limited studies have evaluated a probiotic intervention in NAFLD using probiotic food. A randomized, double-blind, placebo-controlled clinical trial (*n* = 36 NAFLD patients) [87] showed that consuming 300 g/day of probiotic yogurt with *Lactobacillus acidophilus* La5 and *Bifidobacterium lactis* Bb12 for eight weeks reduced the hepatic aminotransferases, total and LDL-cholesterol compared to patients consuming conventional yogurt. Another recent 24-week randomized controlled trial (*n* = 102) [88], investigated the effects of synbiotic yogurt (containing 108 colony-forming units of *Bifidobacterium animalis*/mL and 1.5 g inulin) on NAFLD. Synbiotic yogurt consumption improved the hepatic steatosis (assessed by US) and liver enzyme concentrations. A recent meta-analysis [89] including 1309 patients from 25 studies support the potential use of microbial therapies in the treatment of NAFLD after demonstrating a significant reduction in BMI, liver enzymes, serum cholesterol and triglycerides, however, no changes in inflammation have been reported, and whether these effects can be sustained remains uncertain. Though, in an animal NAFLD/NASH model, probiotics have shown to prevent liver fibrosis even in the absence of significant changes in inflammatory markers and in the liver fat [90]. Currently, there is insufficient evidence to recommend its use, but given their good safety profile (except in immunocompromised patients where a risk of fungemia has been described) and that the described studies support their benefit, we can safely advise its use in NAFLD. Further trials with clinically relevant liver related outcomes would be informative.

#### 4.4.2. Coffee

Evidence-based on observational studies report an inverse association between caffeine intake and NASH [91,92]. Coffee has antioxidant, anti-inflammatory and anti-fibrotic properties that could explain this finding. In a cross-sectional study (*n* = 177) [93], liver biopsy was taken at the baseline and coffee consumption was gathered prospectively for 6 months; a daily coffee consumption (>2 cups/day) was associated with significantly lower odds of liver fibrosis. Similar results were seen two years later in a NASH specific cohort (*n* = 306) [94]. In a further prospective study (*n* = 5147) [95], the coffee consumption was recorded at the baseline and after 7 years in NAFLD patients. Those who drank more coffee (>3/day) had a lower fibrosis score. A 2013 systematic review [92] assessed the liver effects of coffee, showing that its consumption was associated with improved serum GGT, AST and ALT values in a dose-dependent manner. Additionally, coffee consumption was inversely related to the severity of NASH. Moreover, coffee consumption could reduce the risk of developing hepatocellular carcinoma in a 63,257 person cohort [96]. Individuals who consumed 3 or more cups/day experienced a 44% risk reduction of developing hepatocellular carcinoma. Prospective studies are needed to confirm these effects and determine the exact dose capable of inducing histological changes.

#### 4.4.3. Resveratrol

Resveratrol is a dietary antioxidant found in red wine. Nineteen patients were enrolled in a 4-week double-blind randomized study comparing resveratrol (10 mg/day) vs. the placebo [97]. Resveratrol appears to improve insulin sensitivity and oxidative stress. Their effect on a more efficient insulin signaling via the Akt pathway might be a positive influence against liver steatosis. These interventions remain of theoretical interest until well-conducted prospective data and larger clinical trials are available.

#### 4.4.4. Alcohol

A cross-sectional analysis of 251 NAFLD lifetime non-drinkers vs. 331 NAFLD modest drinkers (≤2 drinks/day) found that modest drinkers had lower odds of having NASH, fibrosis or ballooning [98]. Similarly, alcohol consumption was analyzed in 77 biopsy-proven NAFLD patients, some degree of regular alcohol consumption over one’s lifetime vs. the minimal intake appears to have a protective effect on the histological severity [99]. These findings demonstrate the need for prospective studies to establish alcohol consumption recommendations in NAFLD.

#### 4.4.5. Choline

Choline is an essential nutrient found in egg yolks and animal protein [9]. Choline is a phospholipid component of cell membranes that help in assembling VLDL and lipid transport from the liver. Choline’s dietary requirement is modulated by the estrogen status and by genetic variations in specific genes of choline and folate metabolism [9,100]. A Choline deficiency might genetically induce NAFLD by inducing irregular phospholipid synthesis, lipoprotein secretion flaws and oxidative damage due to mitochondrial dysfunction [16]. Human studies are needed to clarify its therapeutic effect in NAFLD.

The different studies evaluating the effect of macronutrient composition in patients with NAFLD are summarized in Table 3.

## 5. Dietary Patterns

Dietary patterns are based on regular food consumption and take into account interactions between diverse nutrients. The Western dietary pattern, which usually combines high saturated fats and fructose intake, has been implicated in the development of NAFLD [101]. In 2013, 995 adolescents’ dietary patterns were analyzed using factor analysis [102]. The Western dietary pattern was associated with an increased risk of NAFLD, particularly in obese adolescents. A low-calorie diet is necessary to accomplish weight loss (<1500 kcal/day) [103]. The most studied protective dietary pattern in NAFLD is the MD [24].

### 5.1. Mediterranean Diet

Since the 1960s, observations have evidenced lower mortality from cardiovascular disease in countries of the Mediterranean region vs. Northern European populations or the United States of America [1,45,104]. The MD is a dietary pattern originally inspired by their traditional lifestyle, characterized by a high consumption of plant-based foods (whole grains, cereals, seeds, nuts, legumes, vegetables and fruits), moderate consumption of protein-source foods (fish, seafood, and poultry), low to moderate red wine consumption, low consumption of meat, milk and dairy products and usually associated with an optimal physical activity. The MD predominantly contains MUFAs from olive oil, a greater ratio of omega-3/omega-6 PUFAs, polyphenols, carotenoids and high-fiber foods.

Patients with metabolic syndrome under the MD have shown an improvement in insulin resistance and inflammatory markers (CRP, IL-6, IL-7 and IL-18) [105]. A 2013 Cochrane systematic review [106] including 11 randomized trials (*n* = 52,044) concluded that the MD may modulate important cardiovascular risk factors (total cholesterol and LDL-cholesterol reduction). In terms of T2D primary prevention, the PREDIMED randomized controlled trial [107] has demonstrated a relative risk reduction among subjects with a high cardiovascular risk, treated with MD + extra virgin olive oil vs. MD + nuts or low-fat diet without energy restrictions. In T2D, a 12-week trial (*n* = 27) with a cross-over intervention of MD vs. regular diet favored MD due to the lower HbA1C, saturated and trans fatty acids plasma levels [108].

Although it is likely that these data may be extrapolated to people with NAFLD, randomized trials examining the MD histologic liver effect are limited. In NAFLD, MD has shown to reduce hepatic fat and improve hepatic insulin sensitivity independent of exercise and weight loss [24]. In a Hong Kong-Chinese population (*n* = 797), a higher consumption of vegetables, legumes and fruits was associated with a reduced likelihood of having NAFLD [109]. Trovato et al. [110] evidenced a significant reduction in liver fat (assessed by US) and HOMA-IR in a single arm MD 6-month trial (*n* = 90 overweight, non-diabetic patients). The effect of MD was independent of other lifestyle changes. Kontogianni et al. [111] (*n* = 73) found that greater adherence to the MD was significantly correlated with a lower degree of insulin resistance, ALT and NAFLD severity. Similarly, Aller et al. [112] explored the potential associations between MD adherence and histological characteristics in 82 patients with NAFLD. Greater adherence to the MD was associated with a lower likelihood of high-grade steatosis and the presence of steatohepatitis. Trovato et al. [101] evidenced adherence to MD, HOMA-IR and BMI as the most independent predictors of fatty liver severity in an observational study involving 532 NAFLD patients vs. 667 healthy controls. The positive effect on liver inflammation and fibrosis has been tested only in a few observational studies and small population studies. Nevertheless, on 2015, the European Association for the Study of the Liver (EASL), European Association for the Study of Diabetes (EASD) and European Association for the Study of Obesity (EASO) recommended the MD for NAFLD treatment based on this moderate quality evidence [1].

A randomized cross-over trial (12 NAFLD-biopsy-proven patients) [113] reported a reduction in hepatic steatosis (measured by 1H MRS) and insulin resistance after following the MD, independently of weight loss. Patients followed the MD during a 6-week crossover to a standard diet (low fat-high carbohydrate) for the same period of time with a 6-week washout period in between. Recently, a randomized controlled single-blinded clinical trial (*n* = 63) [114] investigated the liver effect of MD vs. a Mediterranean lifestyle (exercising at least 30 min/day, optimal sleep duration and mid-day rest) vs. controls. A Mediterranean lifestyle along with weight loss was better, showing significant improvements in ALT levels and liver stiffness. In another recent randomized controlled trial (*n* = 48) [115], steatosis (measured by MR) and cardio-metabolic risk factors were analyzed in patients receiving an MD vs. low-fat diet for 12 weeks. Both diets improve hepatic steatosis to a similar degree, however, the Framingham risk score, total cholesterol, serum triglyceride, and HbA1c reduction was higher in the MD group with an additional higher adherence compared to a low-fat diet.

The MD may not be practical in some countries or subpopulations, still, recommending at least some of its components may also be helpful. The MD was successfully applied to a multiethnic Australian population demonstrating the possibility of translating key elements of the traditional MD to populations outside the Mediterranean region [116]. Long-term trials including an optimal nutritional analysis with liver-related outcomes are needed. Meanwhile, due to its high potential for long-term sustainability and its capacity to prevent cardiovascular events or related diseases, it seems prudent to recommend this dietary approach.

### 5.2. DASH Diet

The “Dietary Approach to Stop Hypertension” (DASH) diet is a dietary pattern similar to the MD but with an emphasis on the low intake of sodium, total fat, saturated fat, cholesterol and added sugars. Although this dietary pattern was primarily designed for hypertension, it has recently shown beneficial effects on NAFLD [117,118], possibly because high blood pressure constitutes a significant predictor for progressive fibrosis [119]. In 2016, a case-control study [117] in 102 patients with NAFLD vs. 204 controls found an inverse association between the DASH diet score and the risk of developing NAFLD. After adjusting for BMI and dyslipidemia, the significance of this relationship disappeared. In the same year, a randomized controlled trial (*n* = 60 overweight NAFLD patients) [118] conducted for 8 weeks demonstrated a significant reduction in body weight, serum triglyceride level, VLDL, liver enzymes and a concurrent improvement in insulin sensitivity, oxidative stress and inflammation biomarkers in the DASH diet group vs. controls who received a calorie-restricted diet. A higher calcium and magnesium intake characterized the DASH diet and may have beneficial effects on insulin sensitivity by decreasing the oxidative activity and restoring anti-oxidative enzymes [120]. More studies are required to determine the features of the DASH diet that are associated with the greatest benefits in patients with NAFLD.

### 5.3. Low Carbohydrate Diet

Low-CHO diets have become a common strategy for weight management and metabolic-syndrome-related conditions. Low-carbohydrate (60–150 g/day) and very low-carbohydrate diets (<60 g) are popular diets known for their short-term (less than 2 weeks) weight loss capacity. Some low-carbohydrate diets (e.g., Atkins diet) limit the carbohydrate intake to 20 g/day but allow unrestricted amounts of fat and protein. When the carbohydrate intake is <50 g/day, ketosis will develop from glycogenolysis.

In 2003, a one-year multicenter controlled trial (*n* = 63 obese patients) [121] randomly assigned patients to either a low-carbohydrate, high-protein and high-fat diet vs. a low-calorie, high-carbohydrate and low-fat diet. A greater weight loss at six months was evidenced in the low-carbohydrate diet, nevertheless, this difference was not significant at 12 months. Furthermore, the low-carbohydrate diet was associated with a greater improvement in HDL-cholesterol and triglyceride concentrations. Moreover, five biopsy-proven NAFLD patients went through a six-month low-CHO ketogenic diet (<20 g/day) that significantly improved histologic steatosis, inflammation and fibrosis [122]. However, these results are inconclusive due to the possibility that this improvement may be due to weight loss.

In 2009, 22 obese patients received a calorie-restricted diet (1100 kcal/day) for 3 months, one group had a low-CHO content (<50 g/day) and the other group had a high-CHO content (>180 g/day) [123]. The decrease of intrahepatic triglyceride (measured by H1 MRS) was similar in both groups. A similar study was performed in 2010; 162 obese patients were randomized in a low-fat diet vs. a low-CHO diet for 3 months, both diets showed an improvement in anthropometric measurements (BMI, weight, fat mass), cardiovascular risk factors (blood pressure, HOMA-IR, triglyceride, LDL and total cholesterol levels) and liver enzymes (ALT, AST, GGT) [124]. Later on, a six-month trial [125] of either low-CHO or low-fat diet in 170 overweight patients showed the same effect on intrahepatic lipid accumulation (assessed by MR), independent of visceral fat loss and changes in insulin sensitivity. On 2016, a meta-analysis including twenty clinical trials (*n* = 1073) [126] supports that both low/moderate fat (≤30% of daily calorie intake) and moderate CHO (≤45% of daily calorie intake) diets have a similar beneficial effect on liver function.

Despite the popularity and the weight loss observed with low-carbohydrate, high-protein, high-fat diet (Atkins), and a poor long-term adherence are perceived. A meta-analysis of five trials (*n* = 447) [127] found that low-carbohydrate diets and low-fat diets are both capable of inducing weight loss for a duration of up to 12 months. However, low-carbohydrate diets are associated with unfavorable changes in total cholesterol and LDL-Cholesterol. They concluded that in the absence of cardiovascular morbidity and mortality, such diets currently cannot be recommended for the prevention of cardiovascular disease. A prospective cohort from the Nurses’ Health Study and Health Professionals’ Follow-up Study [128] examined the mortality of low-carbohydrate diets during 26 years of follow-ups. A low-carbohydrate diet based on animal protein and fat was associated with higher all-cause mortality. Low CHO diet has been associated with electrolyte disturbance, hypotension and cholelithiasis [103,129]. A very low-carbohydrate diet should not be recommended if they are not supervised by qualified medical personnel. Thus far, the available evidence supports calorie-restricted diets independently its CHO amount. Long-term and larger studies comparing low-CHO and low-fat diets are needed in patients with NAFLD/NASH, and most important, to evaluate long-term safety and efficacy.

## 6. Conclusions and Future Perspectives

Lifestyle modifications towards a healthy diet and habitual physical activity are desirable in NAFLD. A 7–10% weight loss and its sustainability is the goal in NAFLD patients. Reduced calorie intake, improved macronutrient composition and increased physical activity may act independently to stop disease progression. Dietary adherence is an important determinant of weight loss sustainability. Therefore, it is essential to provide high-quality and practical highlights of dietary interventions for NAFLD. High consumption of CHO, simple sugars, saturated fats, trans fat, animal protein (red meat), and processed food, and a low fiber intake are associated with NAFLD development. Dietary recommendations should consider energy restrictions and the macronutrient composition should be adjusted according to the MD. However, choosing a customized diet with a macronutrient composition according to an individual’s taste may accomplish better compliance. The CHO intake should be oriented towards a high preference for whole grains and low glycemic index foods. The fat intake should aim at a high MUFAs and omega-3 PUFAs consumption. The protein intake should favor vegetable protein consumption. Prebiotic fiber intake and probiotic enriched yogurt must be recommended to promote a reduced calorie intake and a favorable microbiota, respectively. However, the available evidence for dietary interventions is derived mostly from observational studies. To provide strong evidence for lifestyle interventions in NAFLD patients, longer duration trials of standardized dietary interventions evaluating the effect on fibrosis are needed. Moreover, regarding future perspectives, for the nutritional field, it would be important to try to find mechanistic clues. In this context, more insight into the effect of diverse dietary patterns on adipokines and hepatokines should be performed. Noteworthy is that leptin [130], fibroblasts growth factors [131], serum amyloid A [132] and caveoli [133], among others, have been shown to be elevated in obesity and T2D underlying the characteristic low-grade chronic inflammation and decrease following weight loss.

## Figures and Tables

**Table 1:** The summary of dietary indications for the treatment of non-alcoholic fatty liver disease (NAFLD).

**RECOMMENDED**	
Whole grains	Probiotics ^a^
MUFAs	Resveratrol ^a^
Omega-3 PUFAs	Coffee ^a^
Vegetable protein	Taurine ^a^
Prebiotic fiber	Choline ^a^
**TO BE AVOIDED**	
Simple sugars (fructose)	
Saturated and trans fats	
Animal protein (red and processed meat)	

MUFAs: monounsaturated fats; PUFAs: polyunsaturated fatty acids. ^a^ Insufficient evidence available.

**Table 2:** The macronutrient recommendation for the treatment of NAFLD.

**FATS**			
**Type**	**Food Source**	**Evidence**	**Recommendation**
Saturated fats	Animal products (red meat, butter and dairy products), vegetable oils (coconut and palm oil) and processed foods (sausages, desserts)	A risk reduction of coronary events has been evidenced after replacing saturated fats by PUFAs for at least one year [22]	Its consumption is discouraged
Monounsaturated fats	Olive oil, avocados, nuts and nut oils	They have phenolic compounds that are associated with a lower risk of MS [25]. They have shown a significant reduction in liver fat [29], serum triglycerides and fat mass [30]. Additionally, Hb1Ac improvement has been evidenced in T2D [26]	A moderate consumption is recommended
Polyunsaturated omega-6 fats	Vegetable oils (canola and cottonseed), cereal grains (wheat, corn and rice) and nuts	An excess of omega-6 is related to cardiovascular disease, cancer, inflammatory and autoimmune diseases [31]	Its consumption is discouraged
Polyunsaturated omega-3 fats	Seafood, certain vegetable oils (flaxseed oil) and, to a much lesser extent, eggs and meat	Improvement of liver enzymes [41,46] and triglyceride levels [34,44,46] have been evidenced after omega-3 supplementation. Liver fat reduction is still controversial [41,46]; the WELCOME study could not significantly evidence a liver fat reduction nor a reduction in validated liver fibrosis scores; however, the erythrocyte percentage DHA enrichment using gas chromatography was linearly associated with a decreased liver fat percentage [43]	It is advisable to increase omega-3 intake (omega-6/omega-3 ratio of 1-2/1)
Trans fats	Partially hydrogenated vegetable oil, desserts, cream or solid fats	Its consumption is associated with hyperinsulinemia, liver fat accumulation [21] and severe hepatic necroinflammation [47,48]. HCC development has been observed in animal studies [49]	Its consumption is discouraged
**Proteins**			
**Type**	**Food Source**	**Evidence**	**Recommendation**
Animal protein	Red meat and processed meat (sausages)	Its consumption is associated with NAFLD due to its high sodium content and the presence of preservatives, additives, saturated fats and trans fats [21,62]. Cooking meat at high temperatures for a prolonged period is independently associated with insulin resistance [62]. Its consumption is associated with an increase in mortality of all causes and mortality due to liver diseases [61]	Its consumption is discouraged. Avoid specific cooking methods (fried or grilled well done)
Plant-based protein	Whole grains, cereals, seeds, nuts, legumes, vegetables, soybeans, peas	MD is the pattern of choice in the management of NAFLD [2]. It is characterized by the high consumption of plant-based foods.	Its consumption is recommended
**Carbohydrates**			
**Type**	**Food Source**	**Evidence**	**Recommendation**
Simple carbohydrates	Fructose (soft drinks and fruit juices) and refined carbohydrate (sucrose, honey, syrup)	Its consumption is related to a greater hepatic, skeletal and visceral fat deposition [68], as well as, to a higher fibrosis stage [66], due to the de novo lipogenesis and the excessive growth of bacteria in the small intestine [16]. HCC development has been observed in animal studies [49]	Its consumption is discouraged
Dietary fiber	Non-digestible carbohydrates found in garlic, asparagus, leeks, onions and cereals	Dietary fiber may confer a benefit through the modulation of the microbiota. They have shown body weight reduction, decreased serum aminotransferases and improved glycolipid metabolism [72]	Its consumption is recommended

PUFAs: polyunsaturated fats; MS: metabolic syndrome; T2D: type 2 diabetes; WELCOME: Wessex evaluation of fatty liver and cardiovascular markers in NAFLD with Omacor therapy; DHA: docosahexaenoic acid; HCC: hepatocellular carcinoma; NAFLD: non-alcoholic fatty liver disease; MD: Mediterranean diet.

**Table 3:** The published systematic reviews, meta-analysis, human clinical trials and cross-sectional analysis using a liver biopsy to evaluate the effect of macronutrient composition in NAFLD.

SATURATED FATS					
Author, Year	Study Design	*n*	Intervention	Time of Intervention	Results
**Mozaffarian et al., 2010** [22]	A Systematic Review and Meta-Analysis of 8 randomized controlled trials	13,614 participants	Evaluate studies with increased PUFA consumption as a replacement for SFA and report the incidence of myocardial infarction and/or cardiac death	1–8 years	Consuming PUFAs in place of SFA reduces the occurrence of coronary heart disease events by 19%, corresponding to a 10% reduced coronary heart disease risk (RR = 0.90, 95% CI = 0.83–0.97) for every 5% energy increase of PUFAs
**MONOUNSATURATED FATS**					
**Schwingshackl et al., 2011** [26]	A Systematic Review and Meta-Analysis of 9 randomized controlled intervention trials	1547 patients with an abnormal glucose metabolism and being overweight or obese	Evaluate the effects of diets high in MUFAs vs. diets low in MUFAs in glycemic control of T2D	6–48 months	An improvement in Hb1Ac (weighted mean difference–0.21%, 95% CI −0.40 to −0.02; *p* = 0.03) was evidenced but without improvement in fasting plasma glucose or HOMA-IR. MUFAs consumption should be recommended in T2D
**POLYUNSATURATED OMEGA-3 FATS**					
**Toshimitsu et al., 2007** [38]	Applied nutritional investigation	46 patients (28 with biopsy-proven NASH vs. 18 with simple steatosis)	Dietary habits and nutrients intake were analyzed through detailed questioning by physicians and dieticians	3 consecutive days	A higher intake of simple carbohydrates and lower intake of protein, PUFAs and zinc
**Hartweg et al., 2009** [34]	A Systematic Review of 23 randomized controlled intervention trials	1075 T2D patients with cardiovascular risk factors	Effect of omega-3 PUFAs supplementation on NAFLD (mean dose: 3.5 g/day; mean treatment duration: 8.9 weeks)	4 weeks–8 months	Improved triglyceride (lowered by 0.45 mmol/L (95% CI −0.58 to 0.32, *p* < 0.00001)) and VLDL cholesterol (lowered by −0.07 mmol/L (95% CI −0.13 to 0.00, *p* = 0.04)). May raise LDL cholesterol (non-significant in subgroups). No statistically significant effect on body weight, glycemic control, fasting insulin, total or HDL-cholesterol
**Parker et al., 2012** [41]	A Systematic Review and Meta-Analysis of 9 randomized controlled intervention trials	355 patients given either omega-3 PUFAs or the control treatment were included	Effect of omega-3 PUFAs supplementation on NAFLD (median dose: 4 g/day (range: 0.8–13.7 g/day); median treatment duration: 6 months)	8 weeks–12 months	Improvement in liver fat (−0.97, 95% CI −0.58 to −1.35, *p* < 0.001) and in AST levels (−0.97, 95% CI −0.13 to −1.82, *p* = 0.02), however, substantial heterogeneity was found and an optimal dose was not clarified
**Sanyal et al., 2014** [44]	Phase 2b multicenter, double-blinded, randomized, placebo-controlled trial	243 patients with NASH and NAFLD activity scores >4 (75 receives placebo, 82 low-dosage EA (1800 mg/d), 86 high-dosage EA (2700 mg/d)).	Liver biopsies were collected 2 weeks after the last dose. The primary efficacy endpoint was NAS <3, without worsening of fibrosis; or a decrease in NAS by >2 without the worsening of fibrosis	12 months	No significant histological effects or blood markers improvement. However, with 2.7 g of EA, reduced levels of triglyceride were observed (−6.5 mg/dL vs. an increase of 12 mg/dL in the placebo group; *p* = 0.03)
**Nogueira et al., 2016** [46]	Randomized controlled trial	50 patients with biopsy-proven NASH (23 received placebo (mineral oil), 27 received omega-3 PUFAs (flaxseed oil and fish oil)).	Liver biopsies, plasma biochemical markers of lipid metabolism, inflammation, liver function and plasma levels of omega-3 PUFAs were assessed as a marker of intake at the baseline and after 6 months of treatment	6 months	No histological improvement was seen after a six-month use of flaxseed oil and fish oil despite ALA, EA and triglycerides levels significantly improved. NAS improvement was correlated with increased PUFAs plasma levels
**FRUCTOSE**					
**Abdelmalek et al., 2010** [66]	Cross-sectional study	427 NAFLD patients	Block food questionnaire data were collected within 3 months of a liver biopsy	3 months	Daily fructose ingestion from fruit juice and soft drinks is associated with lower steatosis grade and higher fibrosis stage. In patients >48 years, an association with hepatic inflammation and ballooning was found (*p* < 0.05)
**DIETARY FIBER**					
**Daubioul et al., 2005** [78]	Randomized, double-blinded, crossover study	7 patients with biopsy-proven NASH	Daily ingestion of 16 g of oligofructose or maltodextrin (placebo)	8 weeks	Daily oligofructose ingestion decreases serum aminotransferases and improves insulin levels
**PROBIOTICS**					
**Malaguarnera et al., 2011** [86]	Randomized controlled trial	66 patients with biopsy-proven NASH (33 patients received *Bifidobacterium longum* with fructooligosaccharides and lifestyle modification vs. 33 with lifestyle modification alone)	Analytic assessment at 0, 6, 12, 18, and 24 weeks. Liver biopsies were performed at entry and repeated after 24 weeks of treatment	24 weeks	*Bifidobacterium longum* with fructooligosaccharides and lifestyle modification when compared to lifestyle modification alone, significantly reduces TNF-α, CRP, serum AST levels, HOMA-IR, serum endotoxin, steatosis, and the NASH activity index (*p* ≤ 0.05)
**Maet al.,2013** [73]	Meta-Analysis of 4 randomized controlled trials	134 patients	Assess the efficacy of probiotic therapies in modifying liver function, fat metabolism and insulin resistance	8 weeks–6 months	Probiotics can reduce insulin resistance, liver aminotransferases, total-cholesterol and TNF-α. However, the use of probiotics was not associated with changes in BMI, glucose and LDL-cholesterol
**Loman et al., 2018** [89]	Systematic Review and Meta-Analysis of 25 randomized controlled trials	1309 patients with NAFLD	Systemically review and quantitatively synthesize evidence on prebiotic, probiotic, and synbiotic therapies for NAFLD	1.5–4.3 months	Reduction in BMI (0.37 kg/m^2^; 95%CI: 0.46 to 0.28; *p* < 0.001), liver enzymes (ALT, 6.9 U/L (95%CI: 9.4 to 4.3); AST, 4.6 U/L (95%CI: 6.6 to 2.7); GGT, 7.9 U/L (95%CI: 11.4 to 4.4); *p* < 0.001), serum cholesterol (10.1 mg/dL 95%CI: 13.6 to 6.6; *p* < 0.001), serum cholesterol LDL-c (4.5 mg/dL; 95%CI: 8.9 to 0.17; *p* < 0.001) and triglycerides (10.1 mg/dL; 95%CI: 18.0 to 2.3; *p* < 0.001), however, no changes in inflammation (TNF-α and CRP) were reported
**COFFEE**					
**Saab et al., 2014** [92]	Systematic review of case-control or cross-sectional studies	2723 NAFLD patients	Effects of coffee on liver diseases	-	Coffee consumption was associated with improved serum GGT, AST and ALT values in a dose-dependent manner. Coffee consumption was inversely correlated to NASH severity
**ALCOHOL**					
**Dunn et al., 2012** [98]	Cross-sectional Analysis	582 biopsy-proven NAFLD patients (251 lifetime non-drinkers vs. 331 modest drinkers (≤2 drinks/day))	Evaluate the association between modest alcohol drinking (lifetime drinking history questionnaire) and NASH among subjects with biopsy-proven NAFLD	-	Modest drinkers had lower odds of having a diagnosis of NASH (summary OR 0.56, 95%CI: 0.39–0.84, *p* = 0.002), fibrosis (OR 0.56 95%CI: 0.41–0.77) or ballooning (OR 0.66 95%CI 0.48–0.92)
**Kwon et al., 2013** [99]	Cross-sectional Analysis	77 biopsy-proven NAFLD patients	Determine alcohol consumption effect (lifetime alcohol consumption questionnaire) on NAFLD histological severity	-	Some degree of regular alcohol consumption (≥24 gram-years) vs. minimal intake appears to have a protective effect on NAFLD histological severity (OR 0.26, 95%CI: 0.07–0.97, *p* = 0.04)

PUFAs: polyunsaturated fats; SFA: saturated fats; RR: risk ratio; MUFAs: monounsaturated fats; T2D: type 2 diabetes; HOMA-IR: homeostatic model assessment of insulin resistance; NAFLD: non-alcoholic fatty liver disease; NASH: non-alcoholic steatohepatitis; AST: aspartate aminotransferase; NAS: NAFLD activity score; EA: eicosapentaenoic acid; ALA: alpha-linolenic; TNF-α: alpha tumor necrosis factor; CRP: C reactive protein; BMI: body mass index; ALT: alanine aminotransferase; GGT: gamma-glutamyl transferase; OR: odds ratio.
